# Supplementary material for: The interactome of intact mitochondria by cross-linking mass spectrometry provides evidence for coexisting respiratory supercomplexes
Source: Mol Cell Proteomics. 2017 Dec 8;17(2):216–32. doi: 10.1074/mcp.RA117.000470 (PMC5795388; doi:10.1074/mcp.RA117.000470)
Supplement: Supplemental Data [file supp_RA117.000470_133922_0_supp_23978_fzffwf.zip › spectra_annotation/mito_spectra_annotation/280-1-10-1-13-1.pdf]

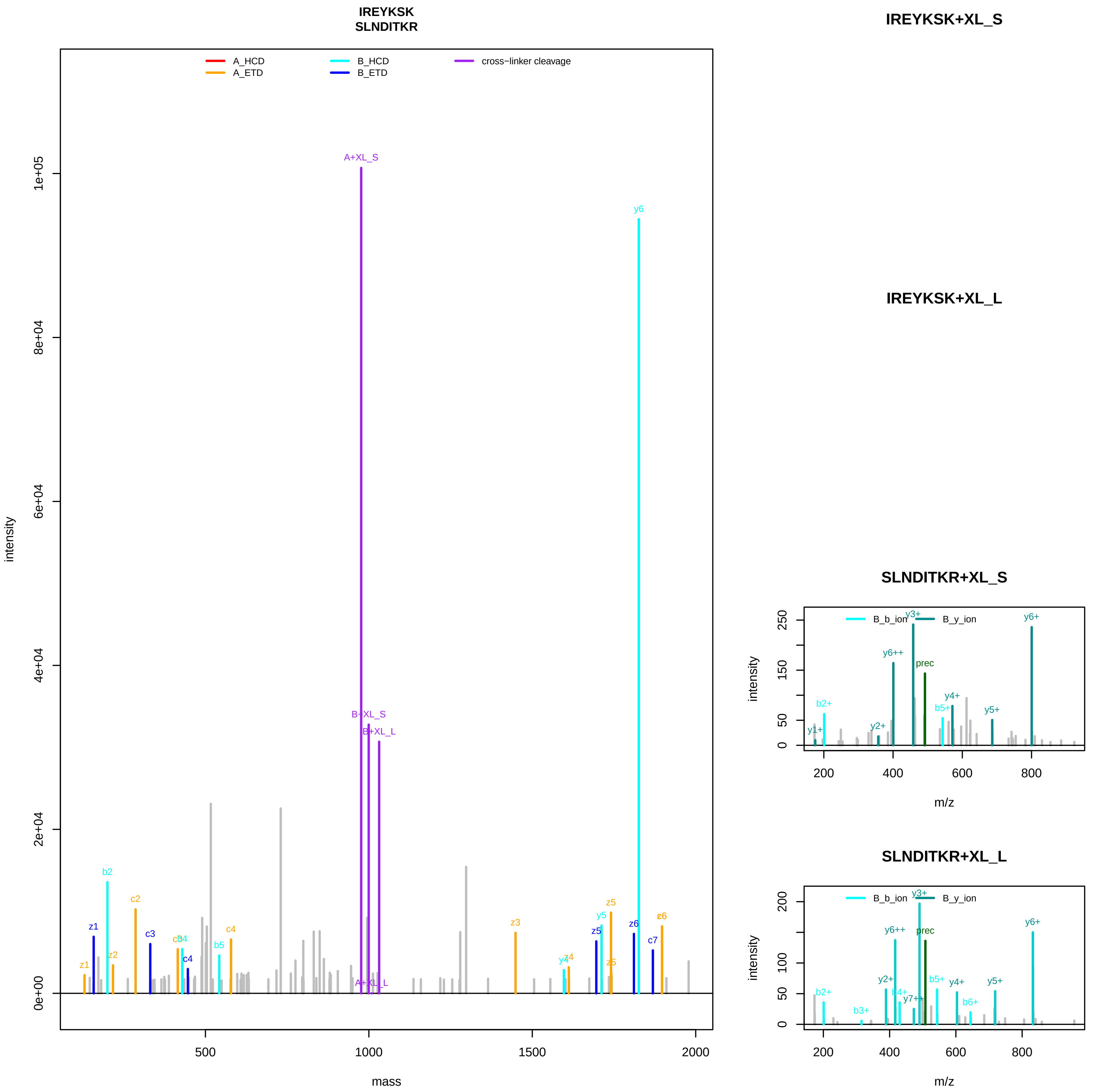

IREYKSK
SLNDITKR
A_HCD
A_ETD
B_HCD
B_ETD
cross−linker cleavage
A+XL_S
B+XL_S
B+XL_L
A+XL_L
y6
b2
z1
c2
z2
c3
c4
b4
b5
c4
z3
y4
z4
y5
z5
z6
c7
z6
intensity
mass
0e+00
2e+04
4e+04
6e+04
8e+04
1e+05
500
1000
1500
2000
IREYKSK+XL_S
IREYKSK+XL_L
SLNDITKR+XL_S
B_b_ion
B_y_ion
y1+
b2+
y2+
y6++
y3+
prec
b5+
y4+
y5+
y6+
intensity
m/z
SLNDITKR+XL_L
b3+
b4+
y7++
b6+
